# Young Adults with Chronic Conditions During the COVID-19 Pandemic: Comparison with Healthy Peers, Risk and Resilience Factors

**DOI:** 10.3390/ijerph22091431

**Published:** 2025-09-14

**Authors:** Ann-Katrin Job, Heike Saßmann

**Affiliations:** 1 Department of Psychology, University of Kassel, Holländische Straße 36-38, 34127 Kassel, Germany; 2 Department of Psychology, Technische Universität Braunschweig, Humboldtstraße 33, 38106 Braunschweig, Germany; 3 Department of Medical Psychology, Hannover Medical School, Carl-Neuberg-Str. 1, 30625 Hannover, Germany; sassmann.heike@mh-hannover.de

**Keywords:** early adulthood, life-long illness, mental health, gender differences, prevention, resilience, risk factors

## Abstract

The COVID-19 pandemic led to psychological impacts for young adults worldwide. Young adults with chronic medical conditions (YACCs) generally experience a higher risk of psychological impairment. This study examined the differences regarding the impact of the pandemic on YACCs compared with healthy peers and aimed to identify risk and buffering factors. A longitudinal survey with *n* = 272 (51% female) young adults was conducted at three time-points during the COVID-19 pandemic. Symptoms of depression and anxiety, positive mental health, life satisfaction, loneliness, and suicidal ideation were assessed, together with sociodemographic variables. The factors contributing to resilient mental health trajectories during the pandemic were examined. A chronic medical condition was reported by 36.8% (*n* = 100) of the participants. Female YACCs, but not male YACCs, experienced significantly more symptoms of anxiety and clinically relevant symptoms of depression more often, and both female and male YACCs reported a significantly lower life satisfaction compared with healthy peers. The corresponding effect sizes were small. YACCs had somewhat higher odds (OR = 1.69) of non-resilient trajectories during the COVID-19 pandemic compared with healthy young adults, although the prediction model explained little variance. The same was true for female compared with male young adults (OR = 1.86). YACCs and female young adults appear to be at higher risk for psychological impairment during pandemic situations. The early detection of those with psychological problems is recommended. Further research is needed to examine the disease-specific influences on resilient trajectories and their interaction with gender and other potentially relevant risk and protective factors.

## 1. Introduction

The COVID-19 pandemic led to a disruption of daily routine, contact restrictions, and limited leisure activities worldwide. The psychological impact of the pandemic, manifested in increased stress, anxiety, and depression levels, was found among adult populations across countries [1,2,3], and among children and adolescents as well as young adults in particular [4,5,6,7]. Young adulthood spans the age range of 18 to the mid/late twenties and is considered an independent developmental phase with high demands regarding independence [8]. Young adults, who generally have an increased risk for mental health problems [9], appeared to be particularly affected by the pandemic [1,5,10,11]. The effects, however, varied: While some young adults reported personal growth during the pandemic, a substantial proportion experienced a deterioration in mental health [12,13]. Regarding children and adolescents (up to 18 years), studies mostly found an increase in psychosocial burden and psychological symptoms during the COVID-19 pandemic [14,15,16].

### 1.1. Risk Factors for Mental Health Problems During COVID-19

Lower socioeconomic status (SES) and female sex were found to be risk factors for increased stress levels in young adults during the pandemic and predictors of reduced well-being [17,18,19]. Having a chronic disease was also found to be a risk factor for a greater psychological impact of the pandemic, both in adolescents and young adulthood [7,14,16,17,20,21,22]. To our knowledge, only one study found no higher risk for young adults with chronic medical conditions (YACCs) [23], which may be due to the small number of participants with chronic illness in that sample (*n* = 49 with chronic illness out of a total sample of 479 participants). Even pre-pandemic YACCs had an elevated risk for mental health problems compared with healthy peers [24,25,26].

### 1.2. Chronic Conditions in Young Adulthood

In a representative German study, 33.8% of females and 25.8% of males aged 18–29 years reported a chronic condition or long-lasting health problem, with higher prevalence rates among individuals with lower educational status [27]. Given the rising incidences of certain chronic diseases worldwide [28] and an elevated risk for poor mental health in YACCs [24,25,26], it seems reasonable to investigate the specific situation of YACCs not only in general, but particularly in times of crises. In this context, it seems important to identify risk factors that may help develop and implement targeted support measures.

The COVID-19 pandemic and its restrictions confronted YACCs with additional challenges, e.g., regarding disease management and clinical appointments. Despite the potential impact of the COVID-19 pandemic on YACCs, to our knowledge there has been no study to date examining the situation of this group in Germany. The present study therefore aims to investigate the short-term effects of the COVID-19 pandemic on psychosocial outcomes in YACCs compared with healthy peers. Since the current study is a longitudinal study with three assessment points at six-month intervals, this study also provides the opportunity to examine differences in the trajectories of mental health problems over the course of one year. Conceptualizing the pandemic as a stressor and because several studies have found an elevated risk for symptoms of depression and anxiety in young adults during the pandemic [4,29,30], we further explore resilience regarding young adults’ psychological well-being. Resilience was operationalized as the absence of clinically relevant depressive and anxiety symptoms over time, whereas any occurrence of clinical levels of anxiety or depression during the observation period was classified as non-resilient [31].

### 1.3. Research Questions and Hypotheses

(1) Are YACCs more affected by the COVID-19 pandemic than healthy peers—regarding symptoms of anxiety and depression, suicidal ideation, and loneliness, as well as positive mental health and life satisfaction? We expected that YACCs would experience a greater psychosocial burden compared with healthy peers.
(2) Are there differences in the mental health trajectories between young adults with and without a chronic condition? We assumed that YACCs would exhibit non-resilient trajectories more frequently than healthy peers.
(3) Which risk and protective factors predict resilient versus non-resilient trajectories in young adults with and without a chronic condition?

## 2. Materials and Methods

### 2.1. Participants and Procedure

The data comes from the German longitudinal study Future Family (FF) and specifically from the 18-year catamnesis of the FF study, Future Family IV (FF-IV), and the additional project Future Family COVID-19 (FF-COVID-19). Both projects were funded by the German Research Foundation (DFG funding codes FF-IV: JO 1632/1-1; FF-COVID-19: JO 1632/3-1). The total FF study started in 2001/02 and examined the effectiveness of the Triple P Positive Parenting Program for families with kindergarten-aged children in two separate studies [32,33]. The initial sample consisted of *N* = 477 families. At the first assessment (Pre) children were on average 4.1 years old (SD = 1.0). The 18-year catamnesis (FU18) of the FF study was conducted between January 2020 to January 2022, when the children had already reached young adulthood. The FF-COVID-19 project was initiated after the outbreak of the COVID-19 pandemic to investigate its impact on the participants of the FF project. Since participants could only be included in the FF-COVID-19 study after the FU18 assessment, the pandemic-related data from the first assessment point (T1) analyzed in the present study were collected between 28 September 2020 (beginning of the 2nd wave of COVID-19 in Germany) and 12 December 2021 (end of the 4th wave according to the Robert Koch Institute, 2022) [34], with two follow-up assessments after 6 months (T2) and 12 months (T3).

Both the FU18 and the FF-COVID-19 assessments consisted of a combination of interviews with the parents and the young adults and standardized questionnaires. Most interviews were carried out by telephone. The questionnaires were completed either using paper–pencil or online via the platform SurveyMonkey. All participants received 50 EUR for participating in the approximately 2.5 h FU18-survey. For participating in the FF-COVID-19 study, the participants received 15 EUR for each of the three assessments and another 5 EUR if they completed all three assessments.

The FF-IV and the FF-COVID-19 studies both received ethical approval from the university IRB board of the University of Braunschweig (identification number FF-IV: D-2019-01; FF-COVID-19: D-2020-09; Faculty of Life Sciences) according to the Declaration of Helsinki. All participants provided written informed consent for study participation and the use of their data for research.

### 2.2. Measurements

The interviews included the acquisition of sociodemographic and clinical information as well as data on leisure and work activities. Standardized questionnaires were used to assess psychosocial burden.

Chronic illnesses. Chronic illnesses were assessed during the FU18-interview. The young adults were asked whether they currently have or had ever had a serious or chronic medical condition. In addition, a list of the following chronic conditions (each with one or two explanatory examples) was queried, covering (1) respiratory diseases, (2) endocrine, nutritional, or metabolic diseases, (3) neurological diseases, (4) skin diseases, (5) rheumatic diseases or other diseases of the muscular or skeletal system, (6) cardiovascular diseases, (7) diseases of the digestive system, (8) tumor diseases, (9) infectious diseases, (10) other pain disorders, (11) other rare or special diseases. Participants indicated the age at diagnosis for each medical condition.

Anxiety symptoms. Self-reported anxiety symptoms were assessed using the Generalized Anxiety Disorder Screener (GAD-7; [35]). The seven items of the GAD-7 inquire on the frequency of symptoms of anxiety and generalized anxiety disorder during the last two weeks according to the Diagnostic and Statistical Manual of Mental Disorders-5 (DSM-5), which are rated on a 4-point scale (0 = “not at all” to 3 = “nearly every day”) and yield a total sum score. Higher scores indicate more symptoms of anxiety during the last two weeks. The internal consistency of the GAD-7 in the current study at T1 was α = 0.86. A total score of ≥10 was used as the cut-off for clinically relevant symptoms of anxiety [36].

Depressive symptoms. The depression module of the *Patient Health Questionnaire*, the PHQ-9 [37], was used to assess self-reported depressive symptoms. The nine items of the PHQ-9 inquire on the frequency of depressive symptoms during the last two weeks, which are also rated on a 4-point scale (0 = “not at all” to 3 = “nearly every day”) and yield a total sum score. Higher scores indicate more depressive symptoms. The internal consistency was α = 0.86 at T1. A total score of ≥10 was used as the cut-off for clinically relevant symptoms of depression [38].

Loneliness. Loneliness was only assessed once, during the last FF-COVID-19 assessment (T3). A single item was formulated and added to the GAD-7 (see above). For this purpose, both the item wording (“During the last two weeks, how often have you been bothered by any of the following problems?—Feelings of loneliness or isolation”) and response scale (0 = “not at all” to 3 = “nearly every day”) were adapted to match the items of the GAD-7. For the analyses, the item responses were dichotomized into 0 = “no loneliness” and 1 = “loneliness on several days to nearly every day”.

Suicidal ideation. The ninth item of the PHQ-9 (see above; [37]) assesses suicidal thoughts during the last two weeks on a 4-point scale from 0 = “not at all” to 3 = “nearly every day”. For the analyses, the item responses were also dichotomized into 0 = “no suicidal thoughts” and 1 = “suicidal thoughts on several days to nearly every day”.

Mental health. Mental health was assessed using the *Positive Mental Health Scale* (PMH; [39]). The nine items capture positive characteristics and behaviors, and are rated on a 4-point scale (0 = “disagree” to 3 = “agree”). The total sum score is calculated, with higher scores reflecting higher levels of positive mental health. The internal consistency at T1 was α = 0.92.

Life satisfaction. General life satisfaction was assessed using the *Life Satisfaction Scale* (LSS; [40]). Respondents report on both the importance of (1 = “not important” to 5 = “very important”) and their satisfaction with (1 = “very poor” to 5 = “very good”) eight different areas of life (e.g., family, friends, living situation, finances, leisure time). Responses are then weighted to calculate a total life satisfaction score. The LSS, including weighted life satisfaction, has been validated in a representative German sample aged 14 to 64 years with norms provided for different age and occupational groups [41]. Higher scores indicate higher life satisfaction. The internal consistency of the LSS total score at T1 was α = 0.74.

### 2.3. Statistical Analyses

Data were analyzed using IBM SPSS Statistics for Windows, Version 29.01 (IBM Corp., Armonk, NY, USA). *χ*^2^-Tests, independent *t*-tests, and multivariate ANOVAs were conducted to examine group differences between YACCs and healthy peers at T1. To examine the trajectories during the COVID-19 pandemic, combined scores for psychological symptoms (PHQ-9 and GAD-7) were created. Since the clinical cut-off of ≥10 of the PHQ-9 and GAD-7 has been validated in representative German studies [36,38], this cut-off was used to classify the mental health trajectories of YACCs and healthy peers as resilient or non-resilient. Those participants with scores under the cut-off (PHQ-9 and GAD-7 < 10) at all three assessment points during the COVID-19 pandemic were classified as resilient, whereas those with psychological symptoms over the cut-off (PHQ-9 or GAD-7 ≥ 10) at any time-point (T1, T2, or T3) were classified as non-resilient. The differences regarding resilient vs. non-resilient trajectories for YACCs compared with healthy peers were examined using *χ*^2^-tests. A stepwise binary logistic regression analysis was then performed to examine the influence of having a chronic condition and other potential risk factors on resilience during the COVID-19 pandemic. The other risk factors considered were sociodemographic variables (biological sex, migration background, highest school leaving certificate, and current occupational status as indicators of the young adults’ socioeconomic background) that have been shown to be important predictors of mental health impairment, in general, as well as during the pandemic in previous studies (e.g., [19,42,43,44]). The interpretation of the effect sizes is based on the usual guidelines: Cohen’s *d* ≥ 0.20 small, *d* ≥ 0.50 medium, *d* ≥ 0.80 large effect; *r* > 0.10 small, *r* > 0.30 medium, *r* > 0.50 large correlation; *η*^2^ > 0.01 small, *η*^2^ > 0.06 medium, *η*^2^ > 0.14 large effect; Cramer’s *Φ* > 0.10 small, *Φ* > 0.30 medium, *Φ* > 0.50 large effect.

## 3. Results

Of the 477 families participating in the initial FF project, *n* = 316 families took part in the 18-year catamnesis (FU18; retention rate: 66.9%). Six families were excluded because they did not meet the inclusion criteria. Of the *n* = 316 families, *n* = 278 young adults also participated in the first assessment of the FF-COVID-19 study (T1 total retention rate: 58.9%). The participation rates for T2 and T3 in the FF-COVID-19 study were *n* = 268 (56.2%) and *n* = 261 (55.2%), respectively. Figure S1 in the Electronic Supplement (ESM) shows the flow of participants through the study. In pre-analyses, no significant differences were found between the young adults whose T1 survey was conducted during the different pandemic phases included. However, *n* = 6 young adults were excluded from further analyses because they were surveyed in other phases of the pandemic and their outcome data differed significantly from those of the other young adults (the detailed results can be requested from the authors).

At T1, the *N* = 272 young adults included in the study had a mean age of 22.3 years (SD = 1.2, range: 20–25). Chronic medical conditions were reported by *n* = 100 (36.8%) young adults. The most frequent diagnoses in the total sample of young adults were thyroid disease (7.7%), asthma (7.4%), atopic dermatitis (7.0%), and migraine (3.7%). The sociodemographic data are summarized in Table 1. There were no significant differences between young adults with and without a chronic medical condition regarding most sociodemographic variables (*p* ≥ 0.149, see Table S1 in the ESM) or in parental participation in the Triple P training in kindergarten age; however, there were significantly more young women in the chronic condition group compared with healthy peers (*χ*^2^ = 14.74; *df* = 1; *p* ≤ 0.001; *Φ* = 0.23). While 25.4% (*n* = 34) of young men reported having a chronic condition, this was the case for almost half of the young women (47.8%; *n* = 66).

Dropout analysis. The young adults that did not take part in FF-COVID-19 study were significantly older (*p* = 0.030; *d* = 0.20) and more likely to have a low family SES (parents’ highest school degree, net household income; *p* ≤ 0.001 in each case; *Φ* = 0.27−0.31) at the start of the FF project in 2001/02. The mothers were more likely to be single parents (*p* ≤ 0.001; *Φ* = 0.19) and reported more severe child behavioral problems (*p* ≤ 0.003; *d* = 0.28−0.30). The representativeness of the present sample is thus limited compared with the original sample.

### 3.1. Differences Between YACCs and Healthy Peers at T1

At the first assessment during the COVID-19 pandemic (T1), YACCs reported significantly more symptoms of depression and anxiety as well as lower positive mental health and general life satisfaction compared with their healthy peers, all with small effect sizes (see Table 2). With regard to the clinical cut-off, 15.0% of the YACCs reported clinically relevant symptoms of anxiety compared with 14.0% of healthy peers. This difference was not significant (*p* = 0.827; *Φ* = 0.01). In contrast, 33.0% of the YACCs reported clinically relevant symptoms of depression compared with 20.5% of healthy peers. This difference was statistically significant but small in magnitude (*p* = 0.022; *Φ* = 0.14). No significant differences were found regarding feelings of loneliness (assessed at T3) and suicidal ideations (see Table 2).

To take the unequal gender distribution in the YACCs and healthy peers groups into account, the comparisons were again carried out separately for young men and women (see Table 3). The results showed that only general life satisfaction (LSS) was significantly lower in YACCs for both men (*p* = 0.046; *d* = 0.33) and women (*p* = 0.020; *d* = 0.36). Regarding anxiety (GAD-7), only female YACCs (*p* = 0.039; *d* = −0.30) reported significantly more symptoms compared with their healthy peers, whereas no group difference was observed among men (*p* = 0.387; *d* = −0.12). Differences in depressive symptoms and positive mental health were non-significant for both young men (PHQ-9: *p* = 0.173; *d* = −0.20; PMH: *p* = 0.393; *d* = 0.08) and young women (PHQ-9: *p* = 0.068; *d* = −0.26; PMH: *p* = 0.073; *d* = 0.25).

Regarding the clinical cut-offs, no significant differences were found between male YACCs and healthy men neither regarding clinically relevant symptoms of anxiety (GAD-7 cut-off: *p* = 0.360; *Φ* = −0.03) nor depression (PHQ-9 cut-off: *p* = 0.178; *Φ* = 0.08). Among female young adults there was also no significant group difference in clinically relevant anxiety (GAD-7 cut-off: *p* = 0.493; *Φ* = 0.00); however, female YACCs reported clinically relevant symptoms of depression significantly more often than healthy young women, but the effect was small (PHQ-9 cut-off: *p* = 0.038; *Φ* = 0.15). No significant differences were found for suicidal ideation (women: *p* = 0.079; *Φ* = 0.12; men: *p* = 0.401; *Φ* = 0.02) or loneliness (assessed at T3; women: *p* = 0.449; *Φ* = −0.01; men: *p* = 0.264; *Φ* = 0.06).

### 3.2. Differences in the Trajectories of Mental Health Problems Between YACCs and Healthy Peers (T1-T2-T3)

Of the *n* = 272 young adults included in this study, only those who completed the GAD-7 and the PHQ-9 at all three assessment points were considered for the analysis of the trajectories of mental health problems over time (*n* = 235; 86.4%). The results of the comparisons of the young adults included and excluded in the longitudinal analyses with regard to sociodemographic data and psychosocial outcomes at T1 are summarized in Tables S2 and S3 in the ESM. Those young adults who were missing at least one questionnaire (*n* = 37; 13.6%) were significantly less likely to have an A-level/high school degree (*χ*^2^ = 28.87; *df* = 1; *p* ≤ 0.001; *Φ* = 0.33), differed regarding their current occupational status (*χ*^2^ = 15.28; *df* = 2; *p* ≤ 0.001; *Φ* = 0.24), and reported more suicidal ideation (*χ*^2^ = 4.53; *df* = 1; *p* = 0.033; *Φ* = 0.13). Thus, the generalizability of the sample for the longitudinal analyses is limited.

**Figure 1:**
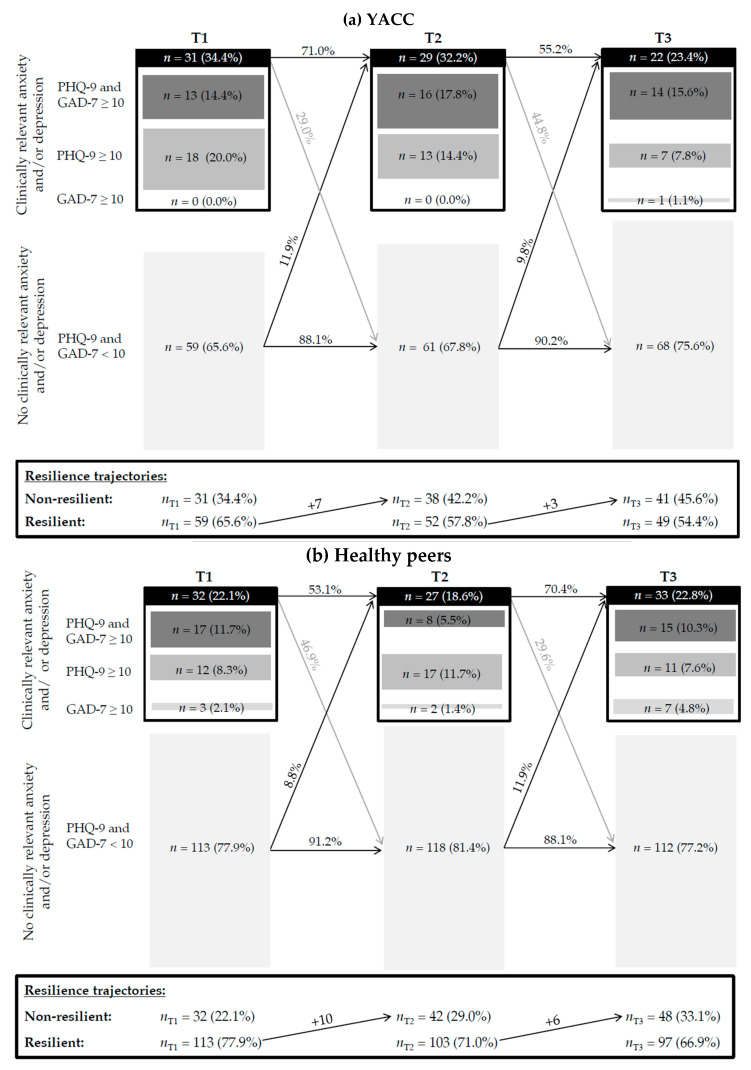
Development of the mental health and the non-resilient trajectories of (**a**) YACCs (*n* = 90) and (**b**) healthy peers (*n* = 145) across the three assessment points according to the GAD-7 and PHQ-9.

Resilient trajectories were significantly more frequent in the group of young adults without chronic conditions, albeit the effect was small (see Table 4). While 33.1% of healthy young adults were categorized as non-resilient, nearly half of the YACCs (45.6%) showed a non-resilient trajectory across the three assessment points, indicating a higher risk of clinically relevant psychological impairment during the pandemic. Figure 1 illustrates the development of the mental health of YACCs and their healthy peers as well as the non-resilient trajectories according to the GAD-7 and PHQ-9 over time.

When conducting the comparison separately for young men and women, no significant difference emerged (men: *χ*^2^ = 0.32; *df* = 1; *p* = 0.288; *Φ* = 0.05; women: *χ*^2^ = 1.82; *df* = 1; *p* = 0.089; *Φ* = 0.12). Descriptive distributions, however, suggest that the group differences in resilience in the total sample were mainly driven by female YACCs (non-resilient: 50.8% vs. 34.4% in male YACCs; see Table S4 in the ESM).

### 3.3. Prediction of Resilient Trajectories in Young Adults

To reduce the likelihood of multicollinearity and number of predictors for the regression analysis, bivariate analyses (*χ*^2^- and *t*-tests) were first conducted for all individual variables (see Table S5 in the ESM). Only sex was significantly associated with resilience (*χ*^2^ = 5.14; *df* = 1; *p* = 0.023; *Φ* = 0.15); all other variables were non-significant (migration background: *p* = 0.0.66; *Φ* = 0.12; current occupational status: *p* = 0.083; *Φ* = 0.05; highest school degree: *p* = 0.421; *Φ* = 0.05; and parental participation in Triple P: *p* = 0.687; *Φ* = 0.03).

Given the significant association between sex and chronic condition in our sample, these variables were examined separately in binary logistic regression analyses with resilience as the dependent variable to determine the variance contribution and odds ratio. The results of both models are shown in Table 5. For chronic conditions, the model only explained 1.5−2.1% of the variance in resilience and did not correctly predict any non-resilient trajectories. Having a chronic condition increased the odds of a non-resilient trajectory by a factor of 1.69. When only sex was included, the model again explained only 2.2−3.0% of the variance, again with no correct classification of non-resilient cases. Female sex increased the odds of a non-resilient trajectory by a factor of 1.86. Although both YACCs and women were more likely to show non-resilient trajectories, the low variance explanations suggest that unmeasured factors are more relevant for predicting resilience during the COVID-19 pandemic.

## 4. Discussion

This is the first study to investigate the psychosocial situation of YACCs during the COVID-19 pandemic in Germany and to compare it with that of healthy peers. The longitudinal data over one year allowed us to examine trajectories within these groups during the pandemic.

More female young adults in our study reported having a chronic condition. This corresponds to the results of earlier studies, even though the difference in our sample was even more pronounced than in the representative German Health Update study [27]. While there was no difference in the percentage of young men reporting a chronic condition (25% vs. 25.8% in our study), significantly more young women in our sample indicated having a chronic condition (33.8% vs. 47.8%). This difference can be explained by the inclusion of a wider variety of chronic conditions in our study (e.g., thyroid disease, which was excluded from the countrywide study). The reason why young women suffer from chronic illnesses more often than men may be that women tend to be more attentive to somatic symptoms and therefore are diagnosed earlier. There is also a general sex difference for some medical conditions, which is in line with our results [45].

### 4.1. Differences Between YACCs and Healthy Peers at T1

In accordance with the majority of the international literature, the YACCs in our sample were more likely to experience depressive symptoms and symptoms of anxiety at the first assessment during the COVID-19 pandemic compared with healthy young adults [16,18]. We additionally explored life satisfaction and mental health, in general, and found YACCs to experience impaired life satisfaction and positive mental health.

In line with previous studies [7,13,14,17,20,21,22], YACCs more frequently reported clinically relevant symptoms of depression (PHQ-9 ≥ 10) compared with healthy peers (33.0% vs. 20.5%). This may reflect the generally lower mental health of YACCs [46] or a more severe impact of the pandemic restrictions. No significant differences, however, were found between YACCs and healthy peers in the percentage of young adults reporting clinically relevant symptoms of anxiety (GAD-7 ≥ 10). Surprisingly, only about 14% of YACCs and healthy peers reported clinically relevant anxiety at T1. This finding is significantly lower than in other COVID-19 studies [4]. While many studies collected their data immediately after the pandemic outbreak, the data collection of the study at hand started in September 2020, i.e., at the beginning of the second wave of COVID-19 in Germany. By this point, the general anxiety levels may already have decreased due to habituation compared with the beginning of the pandemic, which could explain the lower percentage of clinically relevant anxiety in the current sample. A return to lower levels of psychological distress during the same period has also been reported for adults in England [47].

### 4.2. Sex Differences

When the differences were re-examined separately for young men and women, a much more differentiated picture emerged: Most differences were only found in the group of female YACCs but not males. Female YACCs reported significantly more symptoms of anxiety, lower general life satisfaction, and a higher frequency of clinically relevant symptoms of depression compared with healthy young women. In contrast, male YACCs only reported a significantly reduced general life satisfaction compared with healthy young men. Women generally have an increased risk of developing mental health problems compared with men [48]. Several other studies also found a higher impact of the COVID-19 pandemic for the female gender in different age groups [17], suggesting an elevated risk of experiencing impaired mental health and psychosocial problems for girls and women during the pandemic. As these results rely on self-reports, however, it might also be possible that men are less likely to disclose psychosocial problems. Alternatively, male psychological burden may manifest in different symptoms and behaviors than in women, e.g., in a media time rise or alcohol and drug consumption [49]. Other longitudinal studies, however, also found female gender to be a risk factor for impaired mental health during the COVID-19 pandemic. In a study with medical students in China, gender was a main factor associated with mental distress in pandemic times [50]. Another study found the female gender to be associated with a greater deterioration of mental health symptoms across the pandemic among children and adolescents [51].

To explain the higher risk of deteriorated mental health and increased psychosocial burden for females during the pandemic, some authors refer to a potentially stronger impact of social isolation on females, as observed in animal studies [52]. A longitudinal study conducted from the UK reported greater increases in loneliness among women throughout the pandemic [53]. This might also explain the stronger impact of the pandemic on female mental health, even though, at the cross-sectional assessment at T3 in the present study, approximately equal proportions of young men and women reported loneliness (T3). The distribution may have differed at the two earlier assessments when stricter protective measures were in place to contain the pandemic. Other reasons for gender differences include higher pre-pandemic rates of anxiety and depression among women [17] as well as the potential influence of social roles and expectations. For instance, female young adults may have had other domestic responsibilities or felt greater obligation to support family members during times of crisis than young men, increasing their psychological distress [54].

Nevertheless, the results of the current study indicate that differences between YACCs and healthy peers in terms of mental health problems during the COVID-19 pandemic can largely be attributed to young women with chronic conditions. Only general life satisfaction was lower in both young men and women with chronic conditions. Since there is no pre-pandemic data for comparison, however, no conclusions can be drawn as to whether general life satisfaction among YACCs was actually lower due to the pandemic or whether this had already been the case beforehand.

### 4.3. Differences in the Trajectories of Mental Health Problems Between YACCs and Healthy Peers (T1-T2-T3)

Within this longitudinal study we examined young adults with resilient trajectories over one year during the COVID-19 pandemic. Young people with a non-resilient trajectory may be at an increased risk for psychosocial impairment in times of crisis and could therefore benefit from targeted supportive measures. In line with the level of psychosocial burden, we found significantly more non-resilient trajectories in the group of YACCs compared with healthy peers.

Of the potential sociodemographic predictors, only sex showed a significant association with resilience in pairwise comparisons. This result, however, may reflect a sampling effect. When comparing the dropouts to the original FF sample and the FF-COVID-19 sample at T1, young adults without a migration background, with an A-level/high school degree, and who were students were overrepresented in the longitudinal sample. Although there was no significant association between migration background or the young adults’ current occupational status with the resilience trajectories, the effect sizes for both variables were comparable to that for sex. Future research should therefore recruit larger and more heterogeneous samples to clarify the role of additional sociodemographic variables for resilience in times of crisis and potential interactions with sex and chronic medical conditions.

In our study, the regression analyses showed an increased likelihood of a non-resilient trajectory for both YACCs and young women, indicating a higher psychosocial burden during the pandemic. These results confirm previous findings [7,17,18,19]. Although statistically significant, the models explained little variance, suggesting that unmeasured factors are more critical for predicting resilience during the COVID-19 pandemic. For example, trajectories might differ for different medical conditions and more severe chronic diseases could be predictive of non-resilience. Due to the limited sample of YACCs, we were not able to conduct analyses for different chronic conditions. Future studies should therefore examine the specific effects of different chronic diseases. In addition, other factors (e.g., social support, clinical care) may have a stronger influence on the trajectory of YACCs during times of crisis (e.g., [55]).

Our results also suggest that other potential predictors for (non-)resilient trajectories should be investigated in the total group of young adults. These might include social support, environmental factors, and individual variables. Previous studies have shown that, e.g., physical activity [56] and media consumption [57,58], were significantly associated with the mental health of young adults during the pandemic. Such factors may also explain earlier contradictory results regarding the vulnerability of YACCs [23]. Other authors also concluded that although young adults were particularly affected by the pandemic, they are not a homogenous group [59]. They found that subgroups of young adults differed considerably in personality factors and coping strategies.

### 4.4. Strength and Limitations

It is a major strength of this study that young adults with and without chronic conditions were surveyed simultaneously within a relatively large sample. The participants were diverse regarding socioeconomic variables and thus did not represent only a specific subgroup of young adults (e.g., exclusively students). The prospective longitudinal approach furthermore allowed us to examine the trajectories of mental health problems over one year during the pandemic.

There are also several limitations that need to be considered. The results are solely based on self-reports. As we had no access to medical records, some participants may have overestimated their medical problems. Additionally, the assessment time-points were within a certain range, meaning that the restrictions during the pandemic might have differed even within the same wave. The restrictions also varied in different regions of Germany, and some restrictions may have had a stronger impact on young adults’ well-being than others. In this study, only the overall influence of pandemic policies could be investigated. To minimize this impact, pre-analyses examined the potential differences between participants assessed during different phases of the pandemic at T1, with only *n* = 6 excluded from further analyses. Another limitation concerns multiple testing: Given the limited number of YACCs within this study, no Bonferroni’s corrections were applied, which may have increased the risk of alpha error accumulation. Resilience was furthermore only analyzed in a binary way using the clinical cut-offs of the GAD-7 and PHQ-9 for trajectory modeling. This approach does not account for borderline cases and does not allow for interpretations regarding specific disorders. Alternative definitions of resilience, such as improvement, recovery, or even stable symptom levels over time regardless of clinical thresholds, should also be considered in future research [55]. In addition, a distinction was only made between resilient and non-resilient. The non-resilient group, however, also included heterogeneous trajectories (e.g., deterioration, increase, or chronic), which could not be further differentiated due to small subgroup sizes. As another limitation, loneliness and suicidal ideation were each assessed using a single item, which limits the conclusions regarding these constructs. Loneliness was furthermore only assessed at T3, limiting the comparability with the other results. In addition, as no pre-pandemic data were available, it is not possible to determine whether the differences found were actually pandemic-related or already existed beforehand. Since the current sample was originally recruited within an intervention study and nearly half of the sample dropped out over the course of 18 years, the generalizability of the results—particularly of the longitudinal analyses—is limited, which may also be because all of the families initially lived in the same region of Germany. Finally, the sample size of YACCs was relatively small, which may further limit the generalizability of the findings.

Future studies should not only investigate the non-resilient trajectories in more detail but also the impact of other factors on the resilience and psychosocial impairment of young adults during times of crises such as the COVID-19 pandemic. These factors could, e.g., include the sociodemographic variables mentioned above, as well as other factors such as physical activity [56], media consumption (e.g., [57,58]), pre-pandemic mental health problems [60], substance use [61], and adverse childhood experiences [62]. In addition, to better contextualize the findings of the present study, the impact of specific diseases should be analyzed, particularly with regard to gender differences.

Specific information about groups of young adults with a high risk for non-resilient trajectories during crises is essential to inform prevention planning. These groups should be offered specific, evidence-based interventions to reduce psychosocial burden and promote psychological and physical well-being [63]. Preventive interventions should combine established behavioral and cognitive strategies to maintain and improve mental health [63,64]. In particular, mindfulness and exercise practices have shown positive effects on the well-being of young adults during the pandemic [63,65,66].

## 5. Conclusions

This is the first study in Germany examining the psychosocial burden of young adults with and without chronic conditions during the COVID-19 pandemic. Having a chronic medical condition was identified as a risk factor for impaired mental health only in combination with female sex at the first assessment. YACCs were also more likely to show non-resilient trajectories of mental health problems over the course of the pandemic. Both YACCs and young women, in general, had an increased likelihood of a non-resilient trajectory in the prediction model, although the explained variance was very low. Further research is needed to better understand the interaction between chronic medical conditions and gender and to investigate other potential influencing variables. The current findings are important for the clinical care of YACCs. Prevention planning should focus on high-risk subgroups and be maintained throughout crises such as the COVID-19 pandemic to address the needs of those with non-resilient trajectories.

## Figures and Tables

**Table 1:** Sociodemographic data of the sample (*N* = 272).

Characteristic	***n (%)***
Biological sex	
- female	138 (50.7)
- male	134 (49.3)
Migration background	
- no	219 (80.5)
- yes	53 (19.5)
Current living situation	
- lives with at least one parent	190 (69.9)
- already moved out	82 (30.1)
Current intimate relationship	
- no	145 (53.3)
- yes	127 (46.7)
Already have children of their own	
- no	262 (96.3)
- yes	10 (3.7)
Highest school degree	
- no degree to date or lowlevel degree (<10 years of school)	25 (9.2)
- middle level degree (10 years of school)	41 (15.1)
- Alevels/high school degree (13 years of school)	206 (75.7)
Already completed professional training	
- no	186 (68.4)
- yes	86 (31.6)
Current main activity	
- study at a university or college	147 (54.0)
- training/apprenticeship	37 (13.6)
- exclusively professional activity	55 (20.2)
- looking for work	19 (7.0)
- other (school attendance, internship, parental leave, sick leave)	14 (5.1)

**Table 2:** Comparison of psychosocial data between young adults with (*n* = 100) and without (*n* = 172) chronic conditions at COVID-T1.

	Chronic Conditions		*t*	*df*	*p* (1-Tailed)	*d*
	No	Yes				
	*M* (*SD*)	*M* (*SD*)				
Depressive symptoms (PHQ-9)	6.34 (4.60)	7.62 (5.04)	−2.14	269	0.017 *	−0.27
Anxiety symptoms (GAD-7)	4.87 (3.91)	5.91 (4.15)	−2.07	269	0.020 *	−0.26
Positive mental health (PMH)	28.10 (5.61)	26.78 (6.10)	1.82	269	0.035 *	0.23
Life satisfaction (LSS)	53.38 (31.07)	44.52 (33.57)	2.43	266	0.008 **	0.31
	***n* (%)**	***n* (%)**	***χ* ^2^**	***df***	***p*** **(1-tailed)**	***Φ***
Clinically relevant symptoms of anxiety (GAD-7 ≥ 10)			0.05	1	0.414	0.01
- no	147 (86.0)	85 (85.0)				
- yes	24 (14.0)	15 (15.0)				
Clinically relevant symptoms of depression (PHQ-9 ≥ 10)			5.27	1	0.011 *	0.14
- no	136 (79.5)	67 (67.0)				
- yes	35 (20.5)	33 (33.0)				
Suicidal ideation (last 2 weeks)			1.51	1	0.110	0.08
- no	151 (88.3)	83 (83.0)				
- yes	20 (11.7)	17 (17.0)				
Loneliness ^1^ (last 2 weeks)			0.17	1	0.342	0.03
- no	74 (47.7)	41 (45.1)				
- yes	81 (52.3)	50 (54.9)				

** *p* ≤ 0.01; * *p* ≤ 0.05; ^1^ assessed at T3 (one year after T1).

**Table 3:** Young adult men (*n_♂_* = 34) and women (*n_♀_* = 66) with chronic conditions vs. healthy peers (*n_♂_* = 100; *n_♀_* = 71) at COVID-T1.

	Chronic Conditions		*t*	*df*	*p* (1-Tailed)	*d*
	No	Yes				
	*M* (*SD*)	*M* (*SD*)				
Depressive symptoms (PHQ-9)						
- men	6.05 (4.56)	6.94 (5.29)	−0.95	132	0.173	−0.20
- women	6.75 (4.65)	7.97 (4.90)	−1.50	135	0.068 ^#^	−0.26
Anxiety symptoms (GAD-7)						
- men	4.60 (3.97)	4.82 (3.70)	−0.29	132	0.387	−0.12
- women	5.24 (3.93)	6.47 (4.28)	−1.78	135	0.039 *	−0.30
Positive mental health (PMH)						
- men	28.47 (5.24)	28.18 (5.83)	0.27	132	0.393	0.08
- women	27.59 (6.11)	26.06 (6.16)	1.15	135	0.073 ^#^	0.25
Life satisfaction (LSS)						
- men	52.34 (32.15)	40.94 (38.22)	1.69	130	0.046 *	0.33
- women	57.20 (29.51)	46.38 (31.01)	2.08	134	0.020 *	0.36
	***n* (%)**	***n* (%)**	***χ* ^2^**	***df***	***p*** **(1-tailed)**	***Φ***
Men						
Clinically relevant symptoms of anxiety (GAD-7 ≥ 10)			0.13	1	0.360	0.03
- no	89 (89.0)	31 (91.2)				
- yes	11 (11.0)	3 (8.8)				
Clinically relevant symptoms of depression (PHQ-9 ≥ 10)			0.86	1	0.178	0.08
- no	81 (81.0)	25 (73.5)				
- yes	19 (19.0)	9 (26.5)				
Suicidal ideation (last 2 weeks)			0.63	1	0.401	0.02
- no	87 (87.0)	29 (85.3)				
- yes	13 (13.0)	5 (14.7)				
Loneliness ^1^ (last 2 weeks)			0.40	1	0.264	0.06
- no	44 (50.0)	13 (43.3)				
- yes	44 (50.0)	17 (56.7)				
Women						
Clinically relevant symptoms of anxiety (GAD-7 ≥ 10)			0.00	1	0.493	0.00
- no	58 (81.7)	54 (81.8)				
- yes	13 (18.3)	12 (18.2)				
Clinically relevant symptoms of depression (PHQ-9 ≥ 10)			3.16	1	0.038 *	0.15
- no	55 (77.5)	42 (63.6)				
- yes	16 (22.5)	24 (36.4)				
Suicidal ideation (last 2 weeks)			1.98	1	0.079 ^#^	0.12
- no	64 (90.1)	54 (81.8)				
- yes	7 (9.9)	12 (18.2)				
Loneliness ^1^ (last 2 weeks)			0.16	1	0.449	0.01
- no	30 (44.8)	28 (45.9)				
- yes	37 (55.2)	33 (54.1)				

* *p* ≤ 0.05; ^#^ *p* ≤ 0.10; ^1^ assessed at T3 (one year after T1).

**Table 4:** Resilient vs. non-resilient trajectories in young adults with (*n* = 90) and without chronic conditions (*n* = 145).

Trajectory of Psychological Symptoms	Chronic Conditions		*χ* ^2^	*df*	*p* (1-Tailed)	*Φ*
	No	Yes				
	*n* (%)	*n* (%)				
Resilient ^1^	97 (66.9)	49 (54.4)	3.66	1	0.028	0.13
Non-resilient ^2^	48 (33.1)	41 (45.6)				
Total	145	90				

^1^ PHQ-9 and GAD-7 ≤ 10 at T1, T2 and T3; ^2^ PHQ-9 or GAD-7 ≥ 10 at T1, T2 or T3.

**Table 5:** Results of the binary logistic regression analyses to predict non-resilient trajectories according to the GAD-7 and PHQ-9 in young adults during the COVID-19 pandemic.

Independent Variable	B	Wald	*df*	*p*	OR [95–KI]
Chronic condition	0.525	3.63	1	0.057 ^#^	1.69 [0.99−2.90]
	Cox & Snell *R*^2^ = 0.015, Nagelkerkes *R*^2^ = 0.021				
Sex	0.618	5.087	1	0.024 *	1.86 [1.08−3.18]
	Cox & Snell *R*^2^ = 0.022, Nagelkerkes *R*^2^ = 0.030				

* *p* < 0.05; ^#^ *p* < 0.10.

## Data Availability

The datasets of the Future Family project are available upon reasonable request from the corresponding author due to ethical reasons.

## Supplementary Materials

The following supporting information can be downloaded at: https://www.mdpi.com/article/10.3390/ijerph22091431/s1, Figure S1: Flow of participants through the FF-COVID-19 study; Table S1: Comparison of sociodemographic data between YACC and healthy peers (*N* = 272); Table S2: Comparison of sociodemographic data at T1 of young adults that were included in the longitudinal analyses (*n* = 235) and those excluded (after pre-analyses or due to missing PHQ-9 and GAD-7 data at T1, T2, or T3; *n* = 37); Table S3: Comparisons of psychosocial data at T1 of young adults that were included in the longitudinal analyses (*n* = 235) and those excluded (after pre-analyses or due to missing PHQ-9 and GAD-7 data at COVID-T1, T2, or T3; *n* = 36 ^1^); Table S4: Resilient vs. non-resilient trajectories in male and female young adults with and without chronic conditions.; Table S5: Pairwise comparisons of potential sociodemographic predictors for young adults’ resilient trajectories according during the COVID-19 pandemic (*N* = 235).

## Abbreviations

The following abbreviations are used in this manuscript:

GAD-7	Generalized Anxiety Disorder Screener
FF	Future Family
FF-IV	Future Family IV study
FF-COVID-19	Future Family COVID-19 study
FU18	18-year catamnesis/18-year follow-up
LSS	Life Satisfaction Scale
PHQ-9	Depression module of the Patient Health Questionnaire
SES	Socioeconomic status
T1	First assessment of the FF-COVID-19 study
T2	Second assessment of the FF-COVID-19 study
T3	Third assessment of the FF-COVID-19 study
YACCs	Young adults with chronic conditions
