# Supplementary material for: Young Adults with Chronic Conditions During the COVID-19 Pandemic: Comparison with Healthy Peers, Risk and Resilience Factors
Source: Int J Environ Res Public Health. 2025 Sep 14;22(9):1431. doi: 10.3390/ijerph22091431 (PMC12469516; doi:10.3390/ijerph22091431)
Supplement: Supplementary file 1 [file ijerph-22-01431-s001.zip › ijerph-3715139-supplementary.pdf]

Figure S1. Flow of participants through the FF-COVID-19 study.

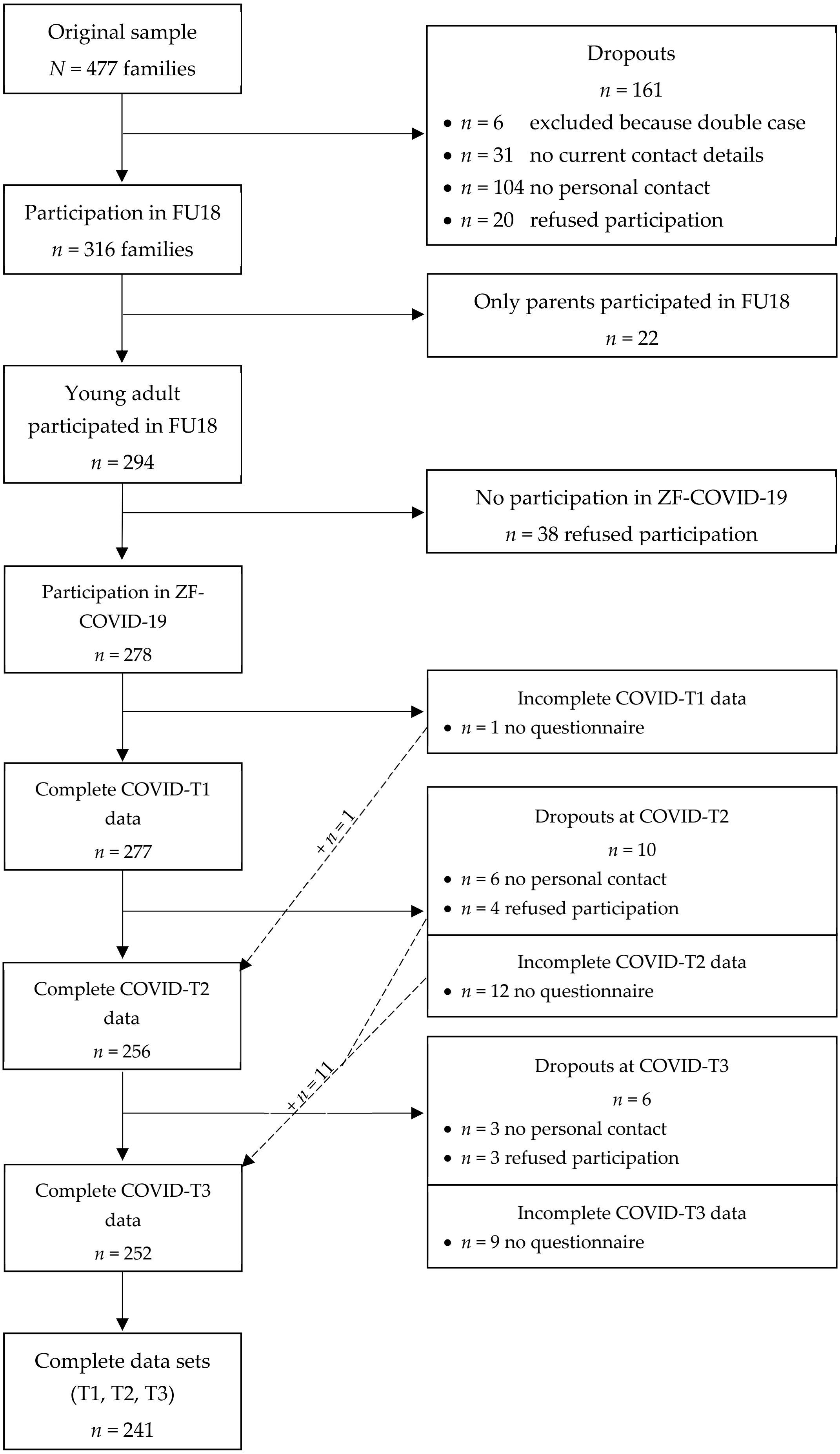

Table S1. Comparison of sociodemographic data between YACC and healthy peers (*N* = 272).

	N	Chronic Condition		$\chi 2$	*df*	*p* (2-tailed)	*Φ*
		**no**	**yes**				
		n (%)	*n* (%)				
Biological sex							
- male	134	100 (74.6)	34 (25.4)	14.74	1	≤0.001***	0.23
- female	138	72 (52.2)	66 (47.8)				
Parental Triple P participation							
- control group	95	54	41	2.57	1	0.109	0.10
- intervention group	177	118	59				
Migration background							
- no	219	138	81	0.02	1	0.878	0.01
- yes	53	34	19				
Highest school degree [1]							
- no degree/low or middle level degree	66	41	25	0.05	1	0.829	0.01
- A-levels/high school degree	206	131	75				
Current living situation							
- with at least one parent	82	53	29	0.10	1	0.753	0.02
- already moved out	190	119	71				
Current occupational status[1]							
- study at a university/college	147	94	53	3.80	2	0.149	0.15
- work or training/ apprenticeship	92	62	30				
- other	33	16	17				
	N	***M* (*SD*)**	***M* (*SD*)**	***t***	***df***	***p* (2-tailed)**	***d***
Age (years)	272	22.28 (1.21)	22.38 (1.14)	−0.64	270	0.524	-0.08

[1] due to small subgroups, some categories were combined; *** $p \leq 0.001$.

Table S2. Comparison of sociodemographic data at T1 of young adults that were included in the longitudinal analyses (n = 235) and those excluded (after pre-analyses or due to missing PHQ-9 and GAD-7 data at T1, T2, or T3; n = 37).

	N	**Included in the Longitudinal Analyses** no	yes	$\chi 2$	*df*	*p* (2-tailed)	*Φ*
		n (%)	*n* (%)				
Chronical condition							
- no	172	27	145	1.75	1	0.186	0.08
- yes	100	10	90				
Biological sex							
- male	134	22	112	1.78	1	0.182	0.08
- female	138	15	123				
Parental Triple P participation							
- control group	96	12	83	0.12	1	0.732	0.02
- intervention group	177	25	152				
Migration background							
- no	219	28	191	0.64	1	0.424	0.05
- yes	53	9	44				
Highest school degree[1]							
- no degree/low or middle level degree	66	22	44	28.87	1	≤0.001***	0.33
- A-levels/high school degree	206	15	191				
Current living situation							
- with at least one parent	82	11	71	0.00	1	0.953	0.00
- already moved out	190	26	164				
Current occupational status[1]							
- study at a university/college	147	9	138	15.28	2	≤ 0.001***	0.24
- work or training/ apprenticeship	92	21	71				
- other	33	7	26				
	N	*M* (*SD*)	*M* (*SD*)	*t*	*df*	*p* (2-tailed)	*d*
Age (years)	272	22.11 (1.31)	22.35 (1.16)	−1.17	270	0.242	−0.21

[1]due to small subgroups, some categories were combined; *** $p \leq 0.001$.

Table S3. Comparisons of psychosocial data at T1 of young adults that were included in the longitudinal analyses (n = 235) and those excluded (after pre-analyses or due to missing PHQ-9 and GAD-7 data at COVID-T1, T2, or T3; n = 36[1]).

	N	**Included in the Longitudinal Analyses**		***t***	***df***	***p* (2-tailed)**	***d***
		**no**	**yes**				
		***M* (*SD*)**	***M* (*SD*)**				
Depressive symptoms (PHQ-9)	271	6.56 (4.44)	6.85 (4.85)	−0.34	269	0.731	−0.06
Anxiety symptoms (GAD-7)	271	6.31 (4.79)	5.09 (3.88)	1.69	269	0.092#	0.30
Positive mental health (PMH)	271	27.08 (5.49)	27.70 (5.89)	−0.60	269	0.552	−0.11
Life satisfaction (LSS)	268	44.30 (34.98)	51.64 (31.89)	−1.22	266	0.223	−0.23
	N	***n* (%)**	***n* (%)**	***χ2***	***df***	***p* (2-tailed)**	***Φ***
Clinically relevant symptoms of anxiety (GAD-7 ≥ 10)				0.17	1	0.676	0.03
- no	232	30	202				
- yes	39	6	33				
Clinically relevant symptoms of depression (PHQ-9 ≥ 10)				0.18	1	0.670	0.03
- no	203	28	175				
- yes	68	8	60				
Suicidal ideation (last 2 weeks)							
- no	234	27	207	4.53	1	0.033*	0.13
- yes	37	9	28				
Loneliness[2] (last 2 weeks)							
- no	115	3	112	1.75	1	0.185	0.08
- yes	131	8	123				

[1] one person less compared to Table E-4 because the T1-questionnaires were missing; [2] assessed at T3 (one year after T1; n = 246); *$p \leq 0.05$; #$p \leq 0.10$.

Table S4. Resilient vs. non-resilient trajectories in male and female young adults with and without chronic conditions.

Trajectory of Psychological Symptoms	Chronic Conditions		$\chi 2$	*df*	*p* (*1-tailed*)	*Φ*
	no	yes				
	n (%)	*n* (%)				
		**Men**				
Resilient [1]	38 (61.3)	30 (49.2)	1.82	1	0.089	0.12
Non-resilient[2]	24 (38.7)	31 (50.8)				
Total	62	61				
		**Women**				
Resilient [1]	59 (71.1)	19 (65.5)	0.32	1	0.288	0.05
Non-resilient[2]	24 (28.9)	10 (34.4)				
Total	83	29				

[1] PHQ-9 and GAD-7 ≤ 10 at T1, T2 and T3; [2] PHQ-9 or GAD-7 ≥ 10 at T1, T2 or T3.

Table S5. Pairwise comparisons of potential sociodemographic predictors for young adults' resilient trajectories according during the COVID-19 pandemic ($N = 235$).

	N	Resilience status[1]		$\chi 2$	*df*	*p* (*2-seitig*)	*Φ*
		resilient	non-resilient				
		n (%)	*n* (%)				
Biological sex							
- male	112	78	34	5.14	1	0.023*	0.15
- female	123	68	55				
Triple P participation							
- control group	83	53	30	0.16	1	0.687	0.03
- intervention group	152	93	59				
Migration background							
- no	146	124	67	3.38	1	0.066#	0.12
- yes	89	22	22				
Highest school degree[2]							
- no degree/low or middle level degree	44	25	19	0.65	1	0.421	0.05
- A-levels/high school degree	191	121	70				
Current occupational status[2]							
- study at a university/college	138	84	54	4.99	2	0.083#	0.15
- work or training/ apprenticeship	71	50	21				
- other	26	12	14				
	N	*M* (*SD*)	*M* (*SD*)	*T*	*df*	*p* (*2-seitig*)	*d*
Age (years)	235	22.4 (1.2)	22.4 (1.1)	0.1	233	0.960	0.01

[1] according to the GAD-7 and PHQ-9 (resilient: T1/T2/T3 < 10; non-resilient: at least one T1, T2, or T3 total score ≥ 10); [2] due to small subgroups, some categories were combined; * $p \leq 0.05$; # $p \leq 0.10$.
